# Photosynthesis at the far-red region of the spectrum in *Acaryochloris marina*

**DOI:** 10.1186/s40659-017-0120-0

**Published:** 2017-05-19

**Authors:** Syed Lal Badshah, Yahia Mabkhot, Salim S. Al-Showiman

**Affiliations:** 1 Department of Chemistry, Islamia College University Peshawar, Peshawar, 25120 Khyber Pakhtunkhwa Pakistan; 2 Department of Biochemistry, Abdul Wali Khan University Mardan, Mardan, 23200 Pakhtunkhwa Pakistan; 3 Department of Chemistry, College of Science, King Saud University, Riyad, Saudi Arabia

**Keywords:** Oxygenic photosynthesis, *Acaryochloris* species, Chlorophyll *d*, Photosystem, Phycobillisomes, P_740_

## Abstract

*Acaryochloris marina* is an oxygenic cyanobacterium that utilizes far-red light for photosynthesis. It has an expanded genome, which helps in its adaptability to the environment, where it can survive on low energy photons. Its major light absorbing pigment is chlorophyll *d* and it has α-carotene as a major carotenoid. Light harvesting antenna includes the external phycobilin binding proteins, which are hexameric rods made of phycocyanin and allophycocyanins, while the small integral membrane bound chlorophyll binding proteins are also present. There is specific chlorophyll *a* molecule in both the reaction center of Photosystem I (PSI) and PSII, but majority of the reaction center consists of chlorophyll *d*. The composition of the PSII reaction center is debatable especially the role and position of chlorophyll *a* in it. Here we discuss the photosystems of this bacterium and its related biology.

## Background

**Fig. 1:**
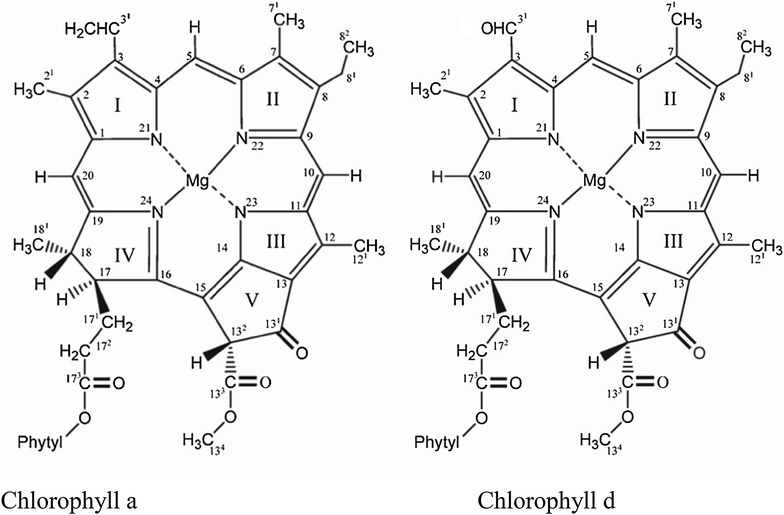
Molecular structure; carbon numbering and specific groups in chlorophyll *a* and *d* molecules

In 1996 a new oxygenic photosynthetic cyanobacterium was discovered, which contains chlorophyll *d* as the major pigment [1]. This organism has a 95% chlorophyll *d* along with 5% chlorophyll *a* in its photosynthetic structures. The main difference between chlorophyll *a* and *d* is the side chain attached at carbon three position of the chlorin ring [2]. In chlorophyll *d*, it is a formyl group instead of a vinyl group (Fig. 1) [1, 2]. Oxidation of the 3-vinyl side chain of chlorophyll *a* to 3-formyl group may leads to the formation of chlorophyll *d* however the enzyme involved is not yet known and it is possibly different from chlorophyllide *a* oxidase (CAO). CAO normally converts *Chl a* to *Chl b*. The ^18^O labeling studies have revealed that the insertion of formyl group in *Chl d* involves an oxygenase enzyme [3]. Further, the oxygen atom in the formyl group of *Chl d* has its origin from molecular oxygen and *Chl a* is the possible biosynthetic antecedent of *Chl d* [3]. There are a number of chemical methods available through which Chl *a* can be converted into *Chl d* without utilizing any enzyme [4, 5]. The presence of this formyl group causes the Qy absorption to red shift by ≈30 nm in vitro [6] and ≈40 nm in vivo [6]. As a result there is 0.1 V lower electronic energy gap between the ground and lower excited state [7, 8]. The molar extinction coefficient of chlorophyll *d* is 63.68 × 10^3^ L mol^−1^ A_697 nm_ cm^−1^ in 100% methanol [9]. The protein binding properties of *Chl d* are similar to that of *Chl a* [10] and that is why *Chl d* can function as both a light harvesting pigment in the antenna as well as a redox active cofactor in the reaction center (RC) of PSI and PSII in *A. marina* [11, 12]. There are few reviews [13–16] available on *A. marina* but still there is a need to further reviewed its photosynthesis as research is going on and there are a number of disagreements on the location of specific cofactors in its reaction centers. The search for the presence of other chlorophyll *d* containing cyanobacteria is also continuous and currently Martinez-Garcia et al. [17] found chlorophyll *d* from the HPLC analysis of phototrophic epibiotic community attached with ascidian *Cystodytes dellechiajei*. However from the denaturing gradient gel electrophoresis they did not find *A. marina* in their bacterial samples. There may be other types of cyanobacteria which may utilize chlorophyll *d* [17]. For example, the production of *Chl d* was recently reported in cyanobacterium called *Chlorogloeopsis fritschii*, when grown under near infra-red light conditions [18]. Sea squirts (ascidians) from the Bahamas showed some new cyanobacteria that have chlorophyll *d* and their 16S rRNA content analysis showed close resemblance with *Acaryochloris* [19]. *A. marina* harvest the far red light with its chlorophyll *d* better [20] and live in symbiotic relationship with other photosynthetic bacteria like Prochloron, Eukaryotic macro-algae and different types of microbial mat in marine environment and also in Salton saline Lake of California where chlorophyll *a* and *b* absorb most of the light [21]. Similarly a new specie has been isolated from the great barrier reef of Australia, which is different in several aspects from the already known species of *Acaryochloris* below crustose coralline algae from endolithic habitat on coral reefs [22]. The search for phototrophic bacteria continued and these bacteria have been isolated from Antarctic rocks and limestones from historical places [23]. They have also been isolated from the coast of Republic of Palau where they live in close association with other cyanobacteria on Didemnid Ascidians and Sponge showing a diversity throughout the world [24]. *Acaryochloris* is now a separate genus that contains seven different strains and the search for new strains is ongoing [25]. These far-red light absorbing cyanobacteria loves to colonize the invertebrate ascidian *Lissoclinum patella* which are present in different layers in shallow water. Thus the ascidian are reservoirs for collection of *A. marina* sample isolation [26]. Kühl and co-workers have developed a new TaqMan-centered quantitative PCR (qPCR) assay for fast screening of environmental samples for identification of *Acaryochloris* species from different geographical locations. The DNA samples of five strains of *A. marina*, i.e., MBIC11017, CCMEE5410, HICR111A, CRS, and Awaji-1, displayed amplification productivities of more than 94%. Further based on this assay, in four samples of crustose coralline algae gathered from moderate and humid regions, *A. marina* was present. Thus this assay is quick and highly efficient method for the detection of *Acaryochloris* strains in diverse locations [25].

## Genomics

*A. marina* is one of the cyanobacterium whose genome has been fully sequenced [27]. There are 8.3 million base pairs (bp) and it is considered as the biggest photosynthetic bacterial genome sequenced so far [27]. Its genomic data is spread into one large chromosome and nine single copy plasmids, which codes for more than 25% open reading frames (ORFs) [27]. This large genome makes *A. marina* a suitable organism to accommodate itself in variable environment with low light conditions. The sequence comparisons showed that these plasmids did not arise from recent duplication phenomena, but some of the plasmids have a good number of sequence area that are homologous. Most of the RNAs are encoded by the large single circular chromosome while the plasmids encoded phycocyanin related genes, hydrogenase genes, genes of the second alternate ATP synthase, and several metabolic enzymes. The *A. marina* genome contains a huge number of genes that help in adaptive regulation of different biological activities. There are a number of *recA* genes which are responsible for repair and recombination enzymes so that the bacterium can expand its genome by duplication of genes and in assimilation of foreign genes. From comparative analysis of several genomes, it has been hypothesized that species with expanded genomes are suitable for living in noncompetitive niche having low but comprehensive amount of nutrients in nature [28]. This hypothesis holds true for *A. marina* as it absorbs at longer wavelength of light which is of no use for other oxygenic photoautotrophs. It fills the noncompetitive niche and is free to develop its metabolic system. An important aspect of *A. marina* genome is the extra genes for alternate ATP synthase on pREB4 plasmid. The role of this ATP synthase is yet to be discovered in the future. The Chl *d* synthase genes are not yet identified. Its genome contains structurally and functionally related homologs for all chlorophyll *a* biosynthesis genes. Different mechanisms of chlorophyll *d* synthesis have been speculated but it may be possible that chlorophyll *d* synthesis chemistry may be different from what is known for chlorophyll chemistry. There are 11 proteins coded by the large chromosomes which are expected to be involved in the biosynthesis of α-carotene and zeaxanthin [27, 29]. The genome has double copies of *crtH, cruA* and *crtQ* but phylogenetic data showed that these genes resemble more to that of filamentous cyanobacteria than for α-carotene of *Prochlorococcus* species. The unique feature of this organism is that it contains genes for both α-carotene and β-carotene biosynthesis, which are *cruA* and *cruP* [27]. Biophysical and biochemical work is needed for the presence of any special interaction between chlorophyll *d* and α-carotene, as *A. marina* exclusively posses α-carotene inside the reaction center. There are two types of accessory light harvesting and a protected method in *A. marina* [27]. These are large number of accessory chlorophyll binding proteins (CBPs) and small amount of phycobiliproteins (PBPs) [30]. The *cpcA* and *cpcG* genes encode phycocyanin and linker proteins which are present on different plasmids. The main chromosomes also contain PBP related genes, these are the three copies of a*pcA* and one copy of *apcB* [27]. The rest of PBP linked genes are located on pREB3 in an enormous number of gene clusters. *A. marina* has a full complement of genes that encode the photosystems [27]. In its sequenced genome, the genes for *psaI* are absent which is important protein for the stabilization of PSI trimer. There are three copies of each *psbA* (D1 subunit) and *psbD* (D2 subunit) of PSII protein genes and are closely homologous to one another [27]. The PSII D1 and D2 subunits primary sequence are extremely well conserved like other cyanobacteria and the only significant difference is that chlorophyll *d* replaces chlorophyll *a* [27]. Further there are duplicated copies of *psbU, psaK* and *psbE* conserved genes on the plasmids of *A. marina*. In *A. marina* genome, most of the cytochrome *b6f* genes are present as a single copy with the exception of *petH* and *petJ* genes [27]. The new *Acaryochloris* species that are isolated from Great Barrier Reef has 2% different 16S rRNA from the ones discovered earlier [19]. The genetic characterization of newly discovered species of *Acaryochloris* are in progress and their genomics show that each species make certain changes in its genome for adaptability to its environment [19]. The *Acaryochloris* sp. HICR111A isolated from waters of Heron Island of Great Barrier Reef enjoys an exceptional genomic section [31]. In this section all the genes for nitrogen fixation are clustered [31]. It is proposed that the genes for nitrogen fixation are possibly transferred through a horizontal gene transfer mechanism among different oxygenic microbes in ocean [31].

The far-red adaptability genes of *A. marina* were recently explored by the use of a high frequency in vivo transposon mutagenesis method that was also successful in other photosynthetic cyanobacteria [32]. Insertion of transposon at various positions in the genome of *A. marina* produced a number of mutants [32]. One of the mutant made through this method produces a yellow colony on agar plates which has the transposon present in the molybdenum cofactor protein A (MoA) gene that disrupted its nitrogen reductase activity. These mutants grow and become functional when either MoA gene is inserted in it or ammonium ion is supplied in the nutrient medium. Thus transposons introduction at various locations are the beginning studies in targeting genes that made *A. marina* suitable for long wavelength photosynthesis [32]. Introduction of chlorophyllide *a* oxygenase (CAO) gene into the genome of *A. marina* resulted into a mutant that accumulates [7-formyl]-chlorophyll *d* molecule [33]. Although the original function of CAO enzyme is to convert *Chl a* to *Chl b*, here it catalyzes the modification of *Chl d* to [7-formyl]-Chlorophyll *d*. This introduction of CAO enzyme expression did not affect the amount of *Chl a* present in *A. marina*, while the amount of the [7-formyl]-chlorophyll *d* is 10% of total chlorophylls present in this species [33]. The [7-formyl]-chlorophyll d is not present in nature. Isolation and purification of PSII showed that there are approximately five [7-formyl]-chlorophyll *d* molecules present in its antenna structure. Biophysical characterization of modified PSII showed that there is transfer of excitation energy from [7-formyl]-chlorophyll *d* to normal chlorophyll *d* while electron transfer in the RC is normal like WT [34].

## Photosystem I

Purified PSI complexes from *A. marina* showed that it has 11 polypeptide subunits that also includes PsaK1 and PsaK2 and also a new smaller subunit called Psa27 [27]. The primary donor (P_740_) of PSI of *A. marina* absorbs maximally at 740 nm and it is a dimer of chlorophyll *d* [35]. PSI of *A. marina* contains one special pair of P_740_; 95–150 chlorophyll *d* molecules, 1 chlorophyll *d* ^/^, 1–2 chlorophyll *a* and about 24–25 α-carotene [36]. Initially it was discovered that the redox mid-point potential (*E*_*m*_) of P_740_ is 335 mV, which is 120–150 mV more negative than P_700_, which has *E*_*m*_ = +420 to +450 mV [2]. However later on it was found that *A. marina* P_740_ of PSI has same redox potential like P_700_ of normal PSI [36, 37]. Due to the presence of *Chl d*, it can absorb energy at longer wavelength than P_700_. The formyl group oxygen may also be hydrogen bonded which affects its absorption properties. Mino et al. [36] identified the oxidized primary donor of PSI through EPR and proton ENDOR spectroscopy. The oxidized special pair (P_740_^+^) has minor differences in *g*-factors values from that of P_700_^+^ of spinach. The hyperfine coupling constants of ENDOR spectrum of oxidized P_700_ is also different from that of P_740_^+^, which are mostly related to the structural differences between Chl *a* and Chl *d* cofactors [36, 38]. It has been speculated that there is a stronger interaction between the two chlorophyll *d* of P_740_ as there is a shift towards red side of the spectrum. It is also expected that the C3 formyl group may act as 6th ligand for the magnesium [7].

Most of the differences are due to the replacement of Chlorophyll *a* by *d* molecule in this special pair. The extinction difference coefficient for the oxidation of primary donor P_740_ is 45,000 ± 4000 M^−1^ cm^−1^ at wavelength of 740 nm [39, 40]. From this extinction coefficient, the calculated ratio of P_740_ to chlorophyll *d* is 1:200 in thylakoid membranes [39]. Chemical composition of the PSI particles from *A. marina* showed that it has α-carotene instead of β-carotene and chlorophyll *d* instead of chlorophyll *a*. There is also similarity between the PsaA and PsaB subunits and it is almost conserved in *A. marina,* as discussed in genomic section. The positions of chlorophylls are also conserved like other oxygenic cyanobacteria. The unusual methionine ligand of ec3 cofactor is different on PsaB subunit and it is replaced by leucine. The spectroscopic analysis of PSI of *A. marina* by Evans and coworkers showed that the functional distance between P_740_ and the phyllosemiquinone is basically identical to those found in other cyanobacteria and land plant systems. Initially, it was suggested that there is a unidirectional electron transfer across the A-branch of the reaction center [41]. However, Santabarbara et al. challenged it and from Nano-second pump-probe spectroscopic studies, they showed that both branches are active. The oxidation of phyllosemiquinones in PSI of *A. marina* showed biexponential decay with a life times of 88 ± 6 and 345 ± 10 ns. Both branches of PSI RC of *A. marina* are active for electron transfer but B-side is slower than that of the PSI of *Synechocystis* [42]. Based on the spectroscopic data, Ohashi et al. have predicted the arrangement of cofactors in the reaction center of photosystems. At position of ec3, which is the primary acceptor of electron, *A. marina* has chlorophyll *a* instead of chlorophyll *d*. The importance of the presence of chlorophyll *a* instead of chlorophyll *d* at ec3 (A_o_) is not yet known. The phylloquinones are the same as that of normal PSI as known from high performance liquid chromatography (HPLC) analysis and mass spectroscopy [7, 14]. The midpoint potential for cytochrome *f* and the primary electron donor of PSI (P_740_) were discovered to be unaltered as compare to other photosynthetic species having Chl *a* and it was 345 mV for Cyt-*f* and 425 mV for P_740_. These results revealed that the midpoint potential of the special pair is not adjusted to the incoming flux of weak excitionic energy in this organism and to conserve the reducing power of PSI [37]. Further investigations revealed that there is no hindrance in the circulation of soluble electron transport protein between cytochrome-*b6f* and PSI in *A. marina*. The chlorophyll fluorescence measurements presented that there is an energy transfer between neighboring PSII complexes but not from PSII to PSI or vice versa. This indicated that there are empty spaces between PSII and PSI [37].

From milli-second photoacoustic (PA) spectroscopy it was observed that the reaction center of photosystem I of *A. marina* absorb more energy as compared to the other cyanobacteria that absorbed at 700 nm wavelength [43]. Thus photosynthesis is not limited by photon of low energy but is possible even at far red light conditions [44]. The excitation energies requirement for P_740_^*^ is 1.68 eV, which is 5% less than that required for P_700_. From photoacoustic data it has been calculated that the efficiency of milli-second time scale photochemistry of *A. marina* is 5–6% higher than that of chlorophyll *a* containing cyanobacteria [44]. The photoacoustic measurement showed that PSI energy trap of *A. marina* is more efficient. The level of maximum efficiency in *A. marina* is 40 ± 1% at 735 nm while it is 35 ± 1% at 690 nm in the *Chl a* containing cyanobacterium *Synechococcus leopoliensis* [45]. During the utilization of near infra-red light for photosynthesis, it was observed that very little reactive oxygen species are produced in different strains of *A. marina*. Thus *A. marina* has adapted its system of photosynthesis where it is free from the stress of reactive oxygen species and free from competing with other cyanobacteria for longer wavelength light [46]. During iron deficiency, the *pcbC* gene is upregulated resulting in the production of the PcbC protein. This PcbC proteins of *Acaryochloris* forms an outer antenna around PSI. In this way they compensate the low level of PSI as compared to the PSII in the *A. marina* cells [47].

## Photosystem II

Ohashi and colleagues proposed sequential arrangements of PSII cofactors in *A. marina* [7]. The P_D1_ and P_D2_ of PSII is debatable and researchers proposed different views about it. These include (1) chlorophyll *a* homodimer, (2) chlorophyll *a/d* heterodimer and (3) chlorophyll *d* homodimer. But chlorophyll d/d^/^homodimer are the most supported one by different experimental techniques [7]. Itoh et al. used electron spin resonance (ESR) technique to measure the Tyrosine D radical (Tyr-D signal) with an X-band ESR in dark adapted thylakoid membranes. The laser excitation induced main bleaching occurs at 725 nm and it decays bi-exponentially with a time constants of 25 μs and 1.2 ms. This P_725_ signal was consigned to the absorption changes that occurred in the special pair *Chl d*; accessory *Chl d* and the acceptor pheophytin *a* of the RC of PSII [41]. Schlodder et al. [48] took flash induced absorbance difference spectrum assigned to the formation of the secondary radical pair of P^+^Q^−^ from 270 to 1000 nm range at 77 K. The 2 ms decay of this spectrum contains features that are similar to *Chl a* molecule. The shape of spectrum near the UV range showed that plastoquinone is the electron acceptor and a strong C550 change at 543 nm indicated that Phe *a* is the primary electron acceptor [48]. The bleaching at 435 nm and absorption at 810 nm showed the signature of *Chl a* and positive charge is stabilized on it [48]. The strong electrochromic band shift at 723 nm was assigned to a neighboring *Chl d* molecule and they suggested that primary charge separation starts here and latter *Chl a* stabilized the positive charge on itself [48]. Thus they are of the view that *Chl a* is present in primary dimeric Chlorophylls of PSII [48]. Renger et al. [49] theoretically analyzed the flash induced difference spectrum of secondary radical pair P^+^Q^−^ of PSII of *A. marina*. They used a modified exciton Hamiltonian for *Chl d* in their calculations. They suggested that there is one *Chl a* molecule at P_D1_ position which acts as a sink for energy and the other is *Chl d* molecule at the P_D2_ site in PSII [49]. Razeghifard et al. studied PSII electron transfer through transient electron paramagnetic resonance (T-EPR) spectroscopy plus chlorophyll fluorescence and low temperature optical spectroscopic methods. They concluded that there are two possibilities that either the dimeric chlorophyll pair of PSII is made of *Chl a* or it is made of *Chl d* but in this case, it is highly modified by the protein environment [50]. Tomo et al. [51] used purified PSII complexes of *A. marina* MBIC 11017 to study the electron transfer events in the RC. There photochemically active PSII particles were composed of a CP47; CP43 (PcbC); D1; D2; cytochrome b559, PsbI and a small polypeptide [51]. The predicted pigment number based on two pheophytin (Phe) a molecules in these purified PSII particle was 55 Chl *d*, 3 Chl *a*, 17 α-carotene and 1 plastoquinone-9 [51]. The light induced difference spectrum at 298 K showed a bleaching at 713 nm and it was assigned to the dimer chlorophylls while a positive peak at 842 nm was assigned to the cation radical of this dimer chlorophylls. From these observations they concluded that dimeric chlorophylls are made of Chl *d* molecules. While there is no signature of chlorophyll *a* at these wavelengths thus the role of chlorophyll *a* in special pair could be excluded [51]. The cation-minus neutral Fourier transform infra-red (FTIR) difference spectrum (1800–1100 cm^−1^) of the special pairs (P^+^/P) in PSII complexes was also significantly different from that of PSII core complexes of spinach and *Synechocyctis* [52]. This FTIR spectrum showed that the dimeric chlorophylls in *A. marina* of PSII contains Chl *d* molecules [52]. Further their photo-accumulation studies confirmed that the primary electron acceptor is pheophytin *a* molecule [52]. Photoacoustic (PA) measurements showed that the trapping efficiency of PSII is 32% at 723 nm for PSII in *A. marina* which is high as compared to a *Chl a* containing PSII from *Synechococcus leopoliensis* having 30% at 671 nm [45]. The proposed energy trap of PSII from PA data fit lies at 723 ± 3 nm that agrees well with other spectroscopic data [45]. These experimental observations also showed that primary charge separation occurs at *Chl d* which is the accessory chlorophyll site (Chl_D1_). Thus the energy trapping efficiency is not limited in *Acaryochloris* and it can perform oxygenic photosynthesis at far red light [45].

The cationic charge distribution over the two chlorophylls of P_D1_/P_D2_ pair of PSII from *A. marina* after electron transfer was computed by using *Chl a* and *Chl d* pairs in different sets. The only pair that showed a non-reliable charge distribution of 50/50 was *Chl d/Chl a* pair set in place of P_D1_^.+^/P_D2_^.+^. It can therefore be predicted that the dimeric chlorophylls of PSII reaction center is not *Chl d/Chl a* pair. The redox potential of Phe *a* at biological conditions of pH 7.0 and temperature of 25 °C is −478 ± 24 mV and estimated redox potential of the special pair is 1.18 V [53]. The redox potential of the quinone molecule *Em* (Q_A_/Q_A_^−^) is +64 mV in the absence of the Mn cluster and by estimation it is between −66 to −86 mV along with the Mn Cluster [53]. Shevela et al. [54] utilized the flash-induced oxygen evolution patterns (FIOPs) to figure out that the redox-potentials of the oxygen evolving complex in its different S states are modified in *A. marina* cells or not. The fitted data in various Kok models showed similar results for both *A. marina* and spinach. Further it was observed that the redox potentials and kinetics inside the OEC, the RC and Y_Z_ are almost same in both *A. marina* and spinach [54]. However there are some minor differences in kinetic and redox values of different S states of OEC in *A. marina* [54]. The free energy ∆G_(PhQ)_ is −325 mV in case of *A. marina* while for *Synechocystis* it is −383 mV [55]. It was generally considered that by harvesting low light, *A. marina* will not be able to break water molecule during electron generation, however the redox potential of this bacteria is 0.1 eV different from that of other cyanobacteria and now it is considered that this will not be a barrier for water oxidation [55]. The light-induced FTIR difference spectra with single reduction of Pheo and Q_A_ presented diverse spectral features in the regions of the keto and ester C=O bands. Similarly the chlorin ring vibrations of Phe and in the CO/CC stretching region of the semiquinone anion Q_A_(−) in *A. marina* also varies from those of the corresponding spectra in Chl *a*-possesing cyanobacteria. Thus in *A. marina* the binding pockets for the Phe and Q_A_ are different in terms of H-bonding interaction as compared to *Chl a* containing cyanobacteria and so is their redox properties [56]. The level of expression of multi-gene families of PSII subunits varies with environmental conditions, especially with different wavelengths of light. It was observed that far-red light enhances the expression of *psbE2* gene that encode the α-subunit of cytochrome b559 and *psbD3* genes that encoded 02′ isoform that is structurally different from normal 02 isoform [57].

An ultra violet and high light intensity of visible and infra-red also causes a decrease in photosystem II activity along with loss of pigments. Similarly as like normal oxygenic photosystem II, UV-B irradiation resulted into fall of manganese from the Mn_4_CaO_5_ cluster of PSII [58].

## Light harvesting system

*Acaryochloris. marina* has two types of antenna system for light harvesting. These include the integral membrane bound accessory chlorophyll binding proteins (CBPs) and the external water soluble phycobiliproteins (PBPs) [7, 30, 59]. In *A. marina* MBIC11017 strain the amount of phycobiliproteins is low as compared to the Awaji-1 strain and due to this reason their fluorescence properties are different, while on the other hand CCMEE5410 strain is devoid of phycobiliproteins [60, 61]. The Cryo-electron transmission microscopy images of whole cell parts showed a number of spots of near crystalline phycobiliprotein at the stromal side of the membrane [30, 37]. These patches of phycobiliproteins separate the reaction centers of the two photosystems from one another [30]. Based on different instrumental data of various workers suggested a model of the antenna of *A. marina* that predicted that PBPs only binds to PSII and not to PSI at non-appressed regions of thylakoid membrane. The model predicts that PSII is externally bound to PBPs and internally with CBPs [30]. In those cyanobacteria which has *Chl a* as a major pigment, for example *Synechococcus* 6301, the excitation energy transfer (EET) from phycobilisomes to PSII showed multiphasic kinetics. Mostly they have lifetimes of 120 ps for the EET from phycocyanin rods to allophycocycnin core then a 70 ps from the core to terminal emitters and final 200 ps lifetime for EET from terminal emitters to *Chl a* of PSII [62]. However in case of *A. marina* the EET from phycobiliprotein aggregates to the *Chl d* of PSII has single exponential kinetics with a lifetime of 70 ± 20 ps that signifies a simple PBP-antenna system. This study confirmed the earlier reports that PBP antenna do not make phycobilisomes but contain rod like aggregates of PC and APC, which directly transfer energy to Chl d without an APC-core as midway junction [62]. The unique collaboration of APC and PC within one hexamer unit of *A. marina* makes a very robust EET from PC to APC, which facilitates a fast EET from phycobiliproteins to PSII with in a time constant of 70 ps. This is three times faster than the EET from terminal emitters of the phycobilisomes to PSII of cyanobacterium *Synechococcus* 6301 [62–64]. Schmitt et al. simulated the excited state population lifetimes data obtained from single photon counting of *A. marina* cells with linear differential equations. The results showed that the EET from terminal emitters of PBP to membrane intrinsic *Chl d* antenna is diffusive in nature and occurs with a time constant of 20–30 ps. From Forster type electronic excitation energy transfer, the calculated distance between terminal emitter of PBP and primary acceptor (*Chl d*) of intrinsic membrane antenna is 2.7 nm for 20 ps transfer step [65, 66]. Incorporation of the accessory chlorophyll binding protein (CBPII) gene *pcbA* of *A. marina* into the genome of *Synechocyctis* PCC6803 showed that it assembled well with the PSII but use chlorophyll *a* as the sole chromophore pigment and these outer CBPIIS transfer energy with high efficiency to PSII while in its presence phycobilisomes are expressed in small amounts [67].

An additional excitation energy pathway in the phycobiliprotein was investigated through transient absorption difference spectroscopy [68]. It was observed that a 14 ps excitation energy pathway exists that runs from phycocyanin to the terminal emitter. This 14 ps excitation pathway is the third and slowest one in addition to the previously reported 300 fs EET that is functional inside the PC trimmer, and 3 ps that transfer excitation energy from hexamer to hetero-hexamer [68]. Difference fluorescence line narrowing (∆FLN) spectroscopy is very useful instrumental technique showing important information about the excitation energy transfer in PBPs of cyanobacteria. The ∆FLN spectrum of *A. marina* PBPs has five low-energy electronic states with fluorescence bands at 635, 645, 654, 659 nm and a last emitter at about 673 nm. The electronic states at 635 represent PC while 645 nm is from APC while the rest of the three bands are possibly linked with three diverse isoforms of the linker protein. The PBPs of *A. marina* is unique due to their highest phonon frequencies as compared to other photosynthetic antenna, in that they range in 31–37 cm^−1^. The associated Huang-Rhys factors S are 0.98 (terminal emitter), 1.15 (APC), and 1.42 (PC) [69]. It is also important to mentioned here that the triplet state of chlorophyll d are formed in the antenna chlorophylls and this Chl *d* has sufficient energy to interact with molecular oxygen, generating singlet oxygen [70, 71].

### Cyanobacteriochromes (CBCRs)

The cyanobacterichromes are the open chain linear tetrapyrrole structure that works as photoreceptors [72]. In *A. marina* there are red/green CBCR AM1_1557, called biliverdin that absorb at longer wavelength as compared to phycocyanobilin (PCB) or phycoviolobilin (PVB) photoreceptors of other cyanobacteria. Thus likes its photosynthetic pigments, its photoreceptor are also red shifted in its absorption spectrum and in that way it helps in its adoption to longer wavelength sun light [73, 74]. The cyanobacteriochromes (CBCRs) of *A. marina* contain a dual-Cys type of CBCR that has reversible photo-conversion with one absorbing at the red-edge of the spectrum with a maximum absorption at 641 nm and it is called the dark state that is inter convertible into the other photoproduct that is absorbing a the blue side with a maximum wavelength of 416 nm. There is also a large difference in wavelength between the two states which is not reported in any other cyanobacteria. Mutational studies showed that there is a covalent linkage formation between the second cysteine and a phycocyanobilin that is reversed when the specie is exposed to blue light.

## Conclusion

Photosynthesis in *A. marina* is unique as it utilizes photons from the far red light part of the spectrum which are low energetically but they are abundant in its habitat where no other photosynthetic organism absorbs such wavelength of light. This long wavelength absorption has been confirmed from UV–visible spectroscopy, EPR, ENDOR, FTIR and other instrumental techniques and growth tests. They required less amount of energy for excitation of their reaction center and so the amount of energy required during electron transfer from oxidation of water to reduce NADP^+^ is smaller. The transfer of energy from extrinsic antenna to membrane bound intrinsic antenna containing chlorophyll *d*, is very efficient and three times faster than other oxygenic cyanobacteria. Although its genome is completely sequenced, but from its expanded genome, the role of different duplicated genes is still to be answered. The position and role of chlorophyll *a* in photosystem II needs to be discovered. In order to fully understand such unique type of photosynthesis there is a need for further spectroscopic and structural based studies of its photosynthetic membrane proteins.

## Abbreviations

APC: allophycocyanin
CAO: chlorophyllide *a* oxidase
CBCRs: cyanobacteriochromes
Chl: chlorophyll
Cyt: cytochrome
CBPs: chlorophyll binding proteins
EET: excitation energy transfer
EPR: electron paramagnetic resonance spectroscopy
*E*_m_: mid-point redox potential
ESR: electron spin resonance spectroscopy
FTIR: fourier transform infra-red spectroscopy
HPLC: high performance liquid chromatography
NADP: Nicotinamide adenine dinucleotide phosphate
OEC: oxygen evolving complex
orf: open reading frames
PA: photoacoustic
PCBs: phycobiliproteins
PC: phycocyanin
PCB: phycocyanobilin
PCV: phycocyanoviolobilin
Phe: pheophytin
PSI: photosystem I
PSII: photosystem II
rRNA: ribosomal ribonucleic acid
UV: ultra-violet radiation
